# Ultrasound as a noninvasive tool for monitoring reproductive physiology in female Atlantic salmon (*Salmo salar*)

**DOI:** 10.14814/phy2.13640

**Published:** 2018-05-06

**Authors:** Ingun Næve, Maren Mommens, Augustine Arukwe, Elin Kjørsvik

**Affiliations:** ^1^ AquaGen AS Trondheim Norway; ^2^ Department of Biology NTNU Trondheim Norway

**Keywords:** Animal welfare, endocrinology, histology, reproduction, ultrasound

## Abstract

Aiming to explore ultrasound technology as a noninvasive method for maturation monitoring, we compared ultrasound observations and measurements in female Atlantic salmon (*Salmo salar*) during the last year before ovulation with standard, invasive methods such as gonadosomatic index (GSI), gonad histology and sex hormone analysis. Ultrasound measurements of ovaries correlated strongly (*R* > 0.9, *P* < 0.01) with ovary weight and GSI, and could be used as a noninvasive tool for GSI estimation. Using ultrasound, we were able to identify females with advanced oocyte development and elevated sex hormone and GSI levels earlier than previously observed. Histological studies confirmed these observations showing oocyte yolk accumulation 10 months before ovulation and 8 months before significant increase in sex hormones. Levels of the sex hormone 11‐keto testosterone (11‐KT) indicated a new role of this hormone at final maturation in salmon females. We propose the use of ultrasound as an alternative method to traditionally used invasive methods during sexual maturation monitoring in wild and farmed Atlantic salmon broodstock populations. Eliminating sacrifice of valuable broodfish, and reducing handling stress, would improve animal welfare in present‐day broodstock management.

## Introduction

Salmonid species are of great economic and nutritional value, and as a consequence their reproduction has been thoroughly studied over decades (Ducharme 1969; Bun Ng and Idler 1978). Triggered by shortening day length and decreasing river temperature, wild Atlantic salmon (*Salmo salar*) spawn in autumn (Heggberget 1988; Webb and McLay 1996). Photoperiod and temperature control enables breeders in commercial Atlantic salmon farming to advance and delay spawning according to market demands for fertilized eggs (Pankhurst and King 2010; Taranger et al. 2010; Vikingstad et al. 2016).

The gonadosomatic index (GSI), gonad weight as percent of body weight, is a measure for maturational status used in fish. Atlantic salmon females typically ovulate and spawn at GSI > 20. In studies covering different aspects of salmonid reproduction, GSI is typically reported in combination with one or more other methods of maturation index, such as sex hormone analysis (Nagahama and Adachi 1985; Prat et al. 1996; Taranger et al. 1998; King and Pankhurst 2003), histology of gonad tissues (Sumpter et al. 1984; Taranger et al. 1999; Estay et al. 2003; Grier et al. 2007), endoscopy (Ortenburger et al. 1996; Swenson et al. 2007) and expression of genes related to sexual maturation in relevant tissues (Campbell et al. 2006; Luckenbach et al. 2008).

GSI estimation, gonad histology, and gene expression studies require fish sacrifice, and while endoscopy and blood sample analysis are less invasive, they might still pose a risk to the health and survival of examined fish (Ortenburger et al. 1996; Swenson et al. 2007) and induce handling related stress (Fast et al. 2008; Eriksen et al. 2015). In addition, most of these methods are limited to provide maturation information on individual fish only once. To monitor maturation in a broodstock population over time, several individuals must be sacrificed, which might not be feasible when working with wild, endangered species or valuable, farmed broodfish. At present, maturation monitoring in wild Atlantic salmon populations relies on GSI measurements requiring sacrifice. In farmed Atlantic salmon, GSI is often estimated from deceased fish, where low fitness may result in a nonrepresentative GSI estimate and maturation status.

Ultrasound technology, a noninvasive technique using echoes from high frequency sound waves to visualize internal organs, has been tested in wild and domesticated fish species, such as European eel (*Anguilla anguilla*; Bureau du Colombier et al. 2015), shovelnose sturgeon (*Scaphirhynchus platorynchus*; Colombo et al. 2004; Bryan et al. 2007; Colombo et al. 2007), sockeye salmon (*Oncorhynchus nerka*; Frost et al. 2014) and striped bass (*Morone saxatilis*; Blythe et al. 1994; Jennings et al. 2005). Ultrasound has been explored as a noninvasive tool for sexing (Colombo et al. 2004; Wildhaber et al. 2005; Guitreau et al. 2012; Frost et al. 2014; Hliwa et al. 2014; Du et al. 2017), basic maturation monitoring (Martin‐Robichaud and Rommens 2001; Moghim et al. 2002; Colombo et al. 2007; Wildhaber et al. 2007; Petochi et al. 2011) and development of advanced methods for estimation of gonad volume, egg size, fecundity and maturity status (Blythe et al. 1994; Bryan et al. 2005, 2007; Jennings et al. 2005; Whiteman et al. 2005; Newman et al. 2008).

Although previous studies indicated the usefulness of ultrasound for maturation monitoring in Atlantic salmon (Mattson 1991), its use has been limited to sex differentiation (Reimers et al. 1987; Mattson 1991) and health inspections (Poppe et al. 1998). We therefore compared ultrasound measurements with traditional invasive methods for maturation monitoring (i.e., GSI, sex hormone analysis and histology). Our goals were to establish (1) a noninvasive method for sexual maturation monitoring in Atlantic salmon using ultrasound technology (2) if ultrasound technology can be an adequate alternative to other invasive methods.

## Materials and Methods

### Ethical approval

All animals were reared and transported according to Norwegian aquaculture legislation. Euthanasia was performed according to Annex IV in European directive 2010/63/EU. In accordance with Norwegian and European legislation related to animal research, formal approval of the experimental protocol by the Norwegian Animal Research Authority (NARA) is not required because the experimental conditions are practices undertaken for the purpose of recognized animal husbandry. The authors have understood the ethical principles the journal operates under, and confirm that this work complies with the checklist provided by Grundy (2015).

### Fish husbandry

Atlantic salmon fry of the AquaGen strain were start‐fed from February 2012 and transferred to net‐pens at a commercial AquaGen sea site in the Hemne fjord (63 °N, 9 °E) in Norway as 1‐year old smolts, in May 2013. During seawater phase, fish were fed according to appetite with Ewos Opal 120 until 1 year before ovulation, when they were fed with Ewos Opal Breed until freshwater transfer. Photoperiod treatment was performed according to commercial broodstock production protocols (Fig. S1). In May 2015, the broodfish were transferred to indoors circular freshwater tanks (8 m diameter, 1.5 m depth, 60 m^3^), and given an autumn light and temperature signal (Fig. S1), until temperature was dropped to induce final maturation and ovulation. Fish were not fed after freshwater transfer as maturing wild Atlantic salmon are not known to feed during spawning migration in rivers (Kadri et al. 1995). Water temperature (Fig. S1) and oxygen levels were recorded daily at 3 and 6 m depth in net‐pens during the seawater phase (58–116% and 75–148%, respectively), and in freshwater tanks (97–114%).

### Experimental design and sampling

Starting in September 2014, 20 females were examined once a month until the temperature drop in August 2015, and then five females were examined weekly until stripping in September 2015. Mean female weight increased from 5.75 ± 0.23 kg in September 2014 to a maximum of 12.56 ± 0.41 kg in April 2015 (see Table S1 for further details). After freshwater transfer, females were not fed, which led to a small nonsignificant decrease in weight (see Table S1). Before sex could be determined by secondary sexual characteristics, ultrasound was used for sexing (see below). Fish were sacrificed with an overdose of tricaine methanesulphonate (200 mg/L, Pharmaq, Norway), followed by spinal transection. Blood was drawn from the caudal vein using heparinized vacuum tubes (Terumo, Japan), and after centrifugation at 2.4 × 1000 *g* for 10 min in 4°C (Micro Star 17R, VWR, USA), plasma was collected and kept on ice for 1–5 h until it could be frozen at −80°C. Body weight was registered to the nearest 20 g using a digital scale (SFE 60K20IMP, Kern & Sohn GmbH, Germany) and fork length to the nearest cm, using a measuring tape. Ultrasound length of left ovary was measured by placing a ruler at the base of the pectoral fin and the ultrasound transducer by the clavicle to locate the anterior tip of the ovary. The transducer was moved in a posterior direction along the ruler until the posterior tip of the ovary was observed in the ultrasound image. Ovary length was measured as distance traveled along the ruler. Cross‐sectional ultrasound images (3–5 depending on ovary length) were captured and compared to histological observations in ovaries. The left ovary was gently removed, and length measured to the nearest mm to verify ultrasound length. Weight of left and both (from December 2014) ovaries was registered to the nearest 0.1 g using a digital scale (MFD, A&D, Japan and Scanvaegt DS‐673SS, Denmark). Cross‐sectional tissue slices, about 0.5–1 cm thick, from the anterior half of the gonad were collected and fixed in 4% formaldehyde solution in phosphate buffer (pH 6.9, Merck Millipore, Germany) for histological analysis.

### Ultrasound specification

A MyLab Alpha ultrasound unit (Esaote, Italy) with a linear array 3–13 MHz probe was used for ultrasound examinations. Ultrasound scanning of fish was performed at 5–7 MHz frequency, with focus point at 25–40 mm, and signal amplification (gain), of approximately 80–90%.

### Sex hormone analysis

Plasma concentrations of sex hormones –estradiol (E2), testosterone (T), 11‐ketotestosterone (11‐KT), follicle‐stimulating hormone (FSH), luteinizing hormone (LH), and maturation inducing hormone (MIH) were analyzed, using enzyme‐linked immunosorbent assay (ELISA) kits from Cayman chemical (Ann Arbor, MI, USA). Frozen plasma samples were thawed on ice and sex hormones were extracted with organic solvent. Briefly, plasma samples were thoroughly mixed with diethyl ether (1:4 plasma:solvent) by vortexing, and the two phases were left to separate. The aqueous phase was frozen in liquid nitrogen, and the sex hormone containing ether phase decanted into a new glass tube before repeating the process. The organic phase was allowed to evaporate overnight and dry extracts were resuspended in 500 *μ*L ELISA buffer, and stored at −80°C until analysis.

Samples were analyzed in duplicate according to the manufacturers protocol, with incubation times of 60 min for MIH, FSH and LH, 70 min for T and E2, and 90 min for 11‐KT. Absorbance readings were performed at 410 nm (11‐KT, E2, MIH and T) and 450 nm (FSH and LH), using a Cytation 5 Imaging plate reader (BioTek Instruments, USA). Standard curves were prepared using Microsoft Excel (Microsoft, USA) by linear regression fit of logit transformed data (E2, T, 11‐KT, MIH) or by 4‐parameter logistic fit, using optical density and standard concentration (FSH, LH, web‐based software: elisaanalysis.com), both as instructed by the manufacturer.

### Histology

Tissue samples for histology were fixed and stored in formaldehyde at 4°C until dehydration in a tissue processor (TP1020, Leica Biosystems Nussloch GmbH, Germany), and then embedded in paraffin. Tissue was sectioned at 4 *μ*m section thickness using a microtome (2055/RM2255, Leica Biosystems Nussloch GmbH, Germany) and stained with standard hematoxylin and eosin (H&E) staining. Microscopy slides were scanned, using a digital slide scanner (NanoZoomer, Hamamatsu photonics, Japan) with up to 40× magnification, and images were exported from the scanner software (NDP, Hamamatsu photonic, Japan) as jpg‐files. Samples from the August and September 2015 samplings were not sectioned as high amounts of lipid rich yolk made this very difficult.

Oocyte development stage was determined according to Taranger et al. (1999) and Andersson et al. (2009; Table S2). Area fraction, area of oocytes in one stage as a fraction of the total tissue area in a section, was determined using a grid plugin for ImageJ (National Institutes of Health, USA). A grid of 231 crosses (Hamilton and Megown In Press, USDA Forest Service Remote Sensing Applications, https://www.fs.fed.us/eng/rsac/invasivespecies/documents/0087-RPT3.pdf) each covering an area of 0.4 mm^2^ was overlaid an image at 1.25× magnification for September 2014 to May 2015 samples, while for June and July 2015 samples, magnification was reduced to 0.7–0.8, while number of crosses in the grid was kept at 231, each now covering 1.1 or 1.2 mm^2^. A cross with center in the tissue qualified as a hit, and was quantified by a cell counter plugin for ImageJ. Oocytes with traits corresponding to more than one developmental stage, were classified according to the most advanced trait. Number of hits within one oocyte stage was multiplied by area covered by each cross, and divided by the total area of the section to calculate area fraction. “Other tissues” accounted for 15–25% of the area throughout the whole sampling period. As the goal was to quantify the area of different oocyte stages in the ovary, it was removed before further calculations were done. Chromatin nucleolus stage oocytes were so small and few that they did not give area fractions, and were excluded from the area fractions figure.

To relate sex steroid levels to oocyte development in addition to sampling time, females were rearranged in four different categories according to their dominating area fraction. Females with area fraction dominated by chromatin nucleolus, perinucleolus, cortical alveoli, and oil droplet stage oocytes were defined as previtellogenic. Females that had started vitellogenesis were divided into three groups; primary, secondary, and tertiary yolk oocyte females (Table S2). Individuals where ovaries contained oocytes in several stages were classified according to the oocyte stage with the highest area fraction.

### Data analysis and statistics

Preparation of data was done, using Microsoft Excel (Microsoft, USA). Figures were made, using Sigmaplot 13 (Systat Software, USA). GSI represents the weight of the gonads as percent of the body weight, and was calculated as: GSI = (gonad weight/body weight)*100. The condition factor, *K*, was calculated as *K* = 100*weight/length^3^.At ovulation, ovarian tissue is degraded, and eggs are released to the abdominal cavity. For fish that had ovulated and were ready to spawn, GSI was defined as zero, as the ovaries could not be dissected and weighed. In September to November 2014, left ovary weight but not total ovary weight was registered. The linear relationship (*R* = 0.99, *P* < 0.001, eq. 1) between left ovary weight (*x*) and total ovary weight (*y*) from December 2014 to March 2015 was used to estimate total ovary weight used to calculate GSI for these samplings.

(1) y=1.74x+1.46

Maturation variation, measured as GSI, was low until March 2015, and the number of samples used for sex hormone analysis and histology was reduced to *n* = 6 per month between September 2014 to February 2015 and 10 per month from March to June 2015, while from July to September 2015, all 20 females were analyzed (Table S1). Statistical analyses were done, using SPSS Statistics 21 (IBM Analytics, USA). All data were tested for normality using Shapiro–Wilk test with significance level of *P* < 0.05 representing nonnormality. Sex hormone (E_2_, T, 11‐KT, LH and MIH) and GSI data were tested for monthly and weekly significant differences using a general linear model (GLM) followed by a least squared difference (LSD) post hoc test to identify significantly different sampling groups. Significance level was set at *P* < 0.05. Data for FSH were not normally distributed, and ANOVA on ranks followed by Dunn's procedure was chosen for this dataset. Significance level was set at *P* < 0.05. All correlations involving GSI measurements and sex hormones were performed, using Pearson product‐moment correlation, with *P* < 0.05. Linear and exponential curves and equations were fitted using regression curve estimation in SPSS Statistics, with *P* < 0.05.

## Results

**Figure 1:**
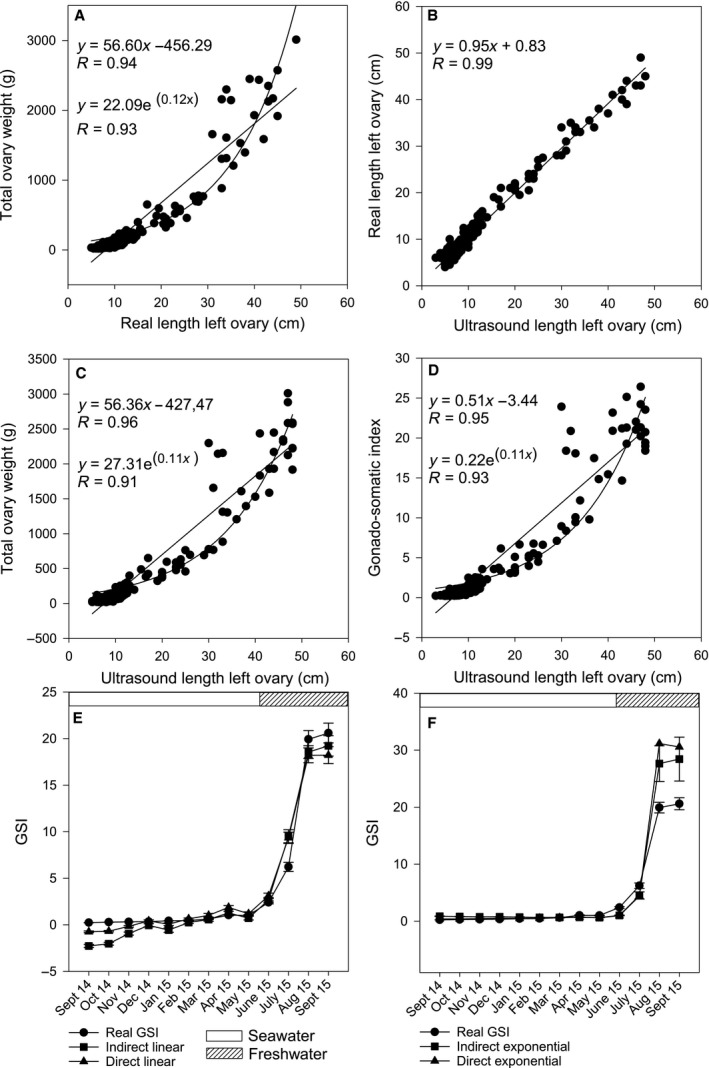
Estimating GSI in Atlantic salmon females from ultrasound measurements. Relationship between real left ovary length and total ovary weight (A) and between ultrasound length and real length of left ovary (B). Linear and exponential models for estimating GSI indirectly by first estimating the total ovary weight and using body weight to calculate GSI (C), or directly from ultrasound length of left ovary (D). Comparison of GSI estimated by linear (E) and exponential (F) method and real GSI of females sampled during the last year before ovulation. Horizontal bars at the top represents changes in environment conditions. Data in E and F are mean ± SEM.

During the seawater phase, mean GSI showed a nonsignificant, gradual increase from 0.23 ± 0.01 to 1.02 ± 0.07 (Fig. 1 in Figs. S2 and S3). After freshwater transfer, mean GSI increased exponentially, and reached a peak of 22.71 ± 1.88 at the end of August 2015, just before ovulation. As final maturation progressed and eggs were released from the ovaries, GSI fell to 0.

There was a strong correlation between left ovary length and total ovary weight (*R* = 0.94, *P* < 0.01, Fig. 1A), and left ovary length measured by ultrasound correlated well with real left ovary length (*R* = 0.99, *P* < 0.01, Fig. 1B). Further, there were strong correlations between left ovary length measured by ultrasound, and both total ovary weight (Fig. 1C) and GSI (Fig. 1D). Linear and exponential equations were fitted, and these can be used to estimate GSI directly from ultrasound length of left ovary, or indirectly by estimating total ovary weight from ultrasound length of left ovary and then calculating GSI, using fish body weight. Measured GSI of females was plotted with GSI estimated using linear and exponential equations given in Figure 1C and D to examine models further (Fig. 1E and F). Both linear models underestimated GSI during the seawater phase and at final maturation. The exponential models were close to measured GSI during the seawater phase when ovary length was small, but overestimated GSI largely at later stages when ovary length increased.

**Figure 2:**
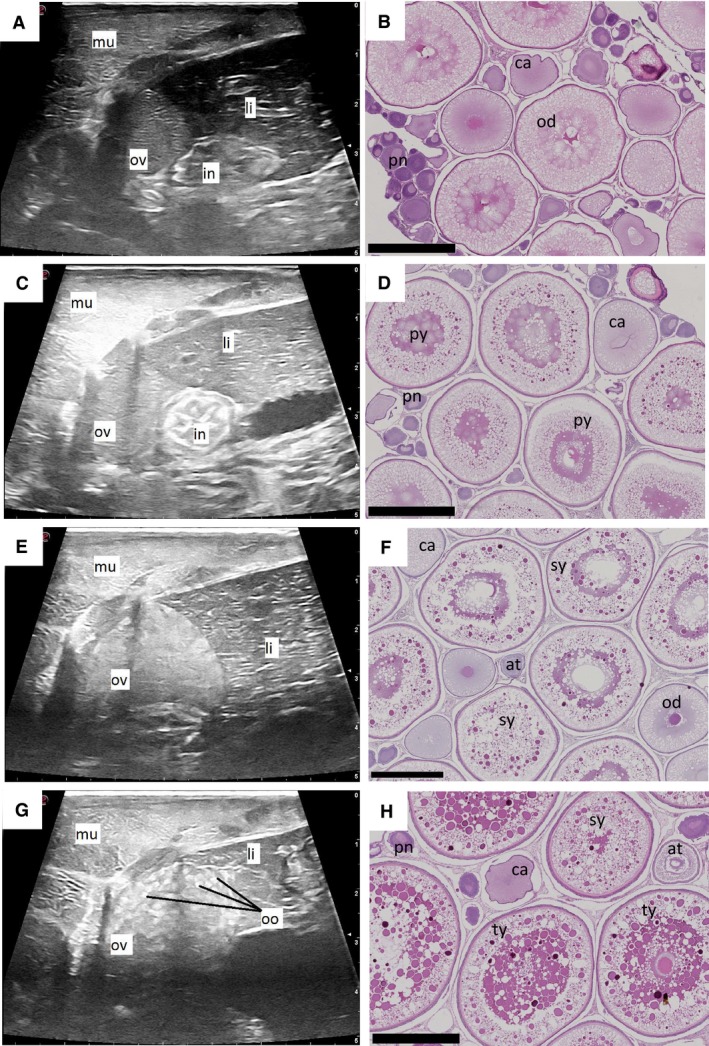
Vitellogenesis in female Atlantic salmon during the last year before ovulation, visualized using ultrasound and histology. Ultrasound (A) and histology (B) of previtellogenic female in September and December 2014, respectively. Ovaries in ultrasound images remain a uniform mass (C) as vitellogenesis has started (D) in some females in Decembler 2014. A growth in ovary area (E) can be observed in ultrasound images as vitellogenesis progresses in February 2015 (F). As some females reach tertiary yolk oocyte stage of vitellogenesis in February 2015, oocytes becomes clearly visible in ultrasound images (G and H). at, atretic oocyte; ca, cortical alveoli stage; in, intenstine; li, liver; mu, muscle; od, oil droplet stage; oo, oocyte; ov, ovary, pn: perinucleolus stage, py: primary yolk oocyte, sy: secondary yolk oocyte, ty: tertiary yolk oocyte. Scalebar in ultrasound images (right side) is 5 cm. Scalebar in all histology slides is 1 mm.

**Figure 3:**
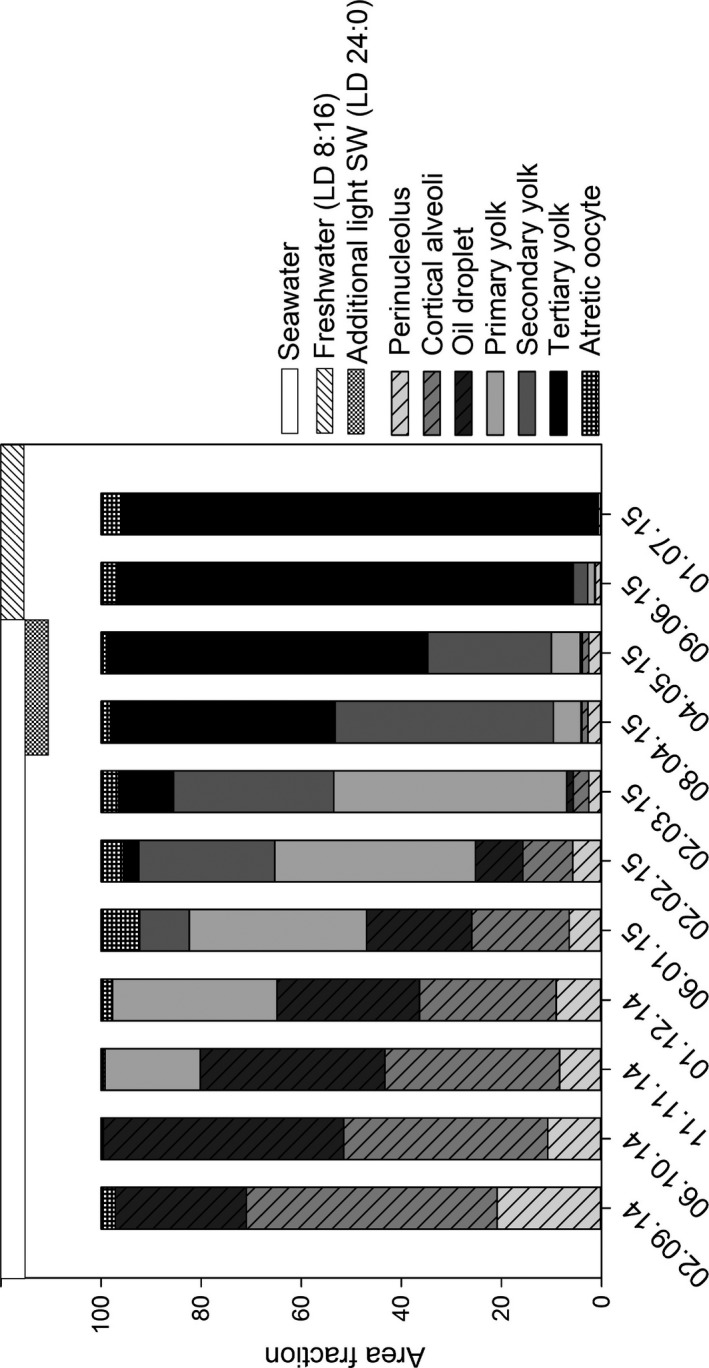
Gonad development of female Atlantic salmon during the last year before ovulation described as area fractions of different oocyte stages in histological sections. The area of oocyte development stages was calculated as percent of total area in sections from 12 to 2 months before ovulation. Horizontal bars at the top represent changes in environment conditions. Data are mean values.

Left ovary cross‐section ultrasound images from each female were examined in relation to histological observations in said females to look for easily discernible structures in the ultrasound images. From September 2014 to January 2015, the ovarian tissue was a uniform mass in the ultrasound image and only a growth in ovary area could be observed (Fig. 2A–E).11 In September 2014, oocytes were predominantly previtellogenic, (Figs. 2A–B and 3) while in January 2015, vitellogenesis had started and ~40% of the observed oocytes were in primary and secondary yolk stage (Figs. 2C–F and 3). In February 2015, one female with tertiary yolk oocytes was observed histologically, and this was the first female where oocytes were discernible in ultrasound images (Fig. 2G–H and 3). In the following months, the occurrence of discernible oocytes observed in ultrasound images increased and were all confirmed to be females with tertiary yolk oocytes. By May 2015, mean area fraction of tertiary yolk oocytes had increased from 3 to 64 % while primary yolk oocytes were reduced to 6%. After freshwater transfer, this development continued, and ended at a total dominance of 95% tertiary yolk oocytes in July 2015 (Fig. 3).

**Figure 4:**
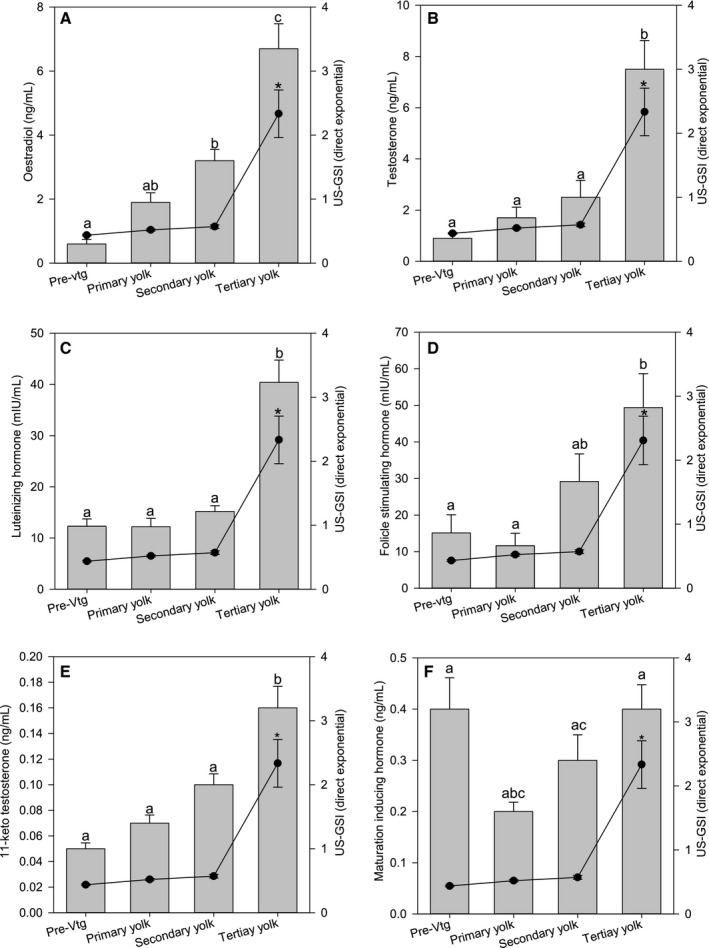
Sex hormone levels in female Atlantic salmon grouped according to dominating oocyte stage area fraction during maturation. Oestradiol (A), testosterone (B), luteinizing hormone (C), 11‐keto testosterone (E) and maturation inducing hormone (F): pre‐vtg; previtellogenic females (*n* = 21; September 2014–February 2015), primary yolk oocytes (*n* = 17; November 2014–March 2015), secondary yolk oocytes (*n* = 14; January–May 2015), tertiary yolk oocytes (*n* = 44; March–July 2015). Follicle stimulating hormone (D): Pre‐vtg: previtellogenic females (*n* = 13; September 2014–January 2015), primary yolk oocytes (*n* = 12; December 2014–March 2015), secondary yolk oocytes (*n* = 14; January–May 2015), tertiary yolk oocytes (*n = *43; March–July 2015). Bars are mean values and S.E.M. The line represents mean ultrasound estimated GSI and S.E.M. GSI was estimated from ovary length using direct exponential approach (Fig. 1D). Letters indicate significant differences in sex hormones, *P* < 0.05. Asterisks represents significant different US‐GSI measures.

Linear models for ultrasound‐based GSI measurements correlated stronger with sex hormone levels (Fig. 2 in Figs. S2 and S3) than exponential models, with the exception of MIH (Table 1). Highest correlations (*R* = 0.74–0.81) were found between ultrasound‐based GSI and levels of 11‐KT, independent of model type. When females were rearranged according to the dominating oocyte stage based on area fractions, the presence of vitellogenic oocytes concurred with a significant increase in sex hormone levels (Fig. 4). Mean plasma levels of E2 increased from those seen in previtellogenic females to those with secondary and tertiary yolk stage oocytes (Fig. 4A). T levels were also significantly higher in females with tertiary yolk oocytes than females with previtellogenic, primary and secondary yolk oocytes (Fig. 4B). Plasma levels of LH were similar in females with previtellogenic, primary and secondary yolk oocytes, but doubled in tertiary yolk oocyte females (Fig. 4C). FSH levels were higher in secondary and tertiary yolk oocyte females than in previtellogenic and primary yolk oocyte females (Fig. 4D). Levels of 11‐KT was low in females with previtellogenic, primary and secondary yolk oocytes, but were significantly higher in tertiary yolk oocyte females (Fig. 4E). The pattern described for the other hormones was not observed for MIH as plasma levels dropped from previtellogenic females to females with primary yolk oocytes and then increased in secondary and tertiary yolk oocytes (Fig. 4F). Ultrasound estimated GSI (direct exponential approach) was low in females with previtellogenic, primary, and secondary yolk oocytes (0.44, 0.52 and 0.57, respectively), while it was significantly higher (2.33, *P* < 0.05) in females with ovaries dominated by tertiary yolk oocytes (Fig. 4).

**Table 1:** Correlations of sex hormone levels to ultrasound‐based GSI estimates and real GSI of Atlantic salmon females during the last year before ovulation. Numbers are *R*,* P* < 0.01 for all correlations. 11‐KT, 11‐keto testosterone; E2, oestradiol; FSH, follicle‐stimulating hormone; GSI, gonado‐somatic index; LH, luteinizing hormone; MIH, maturation inducing hormone, T, testosterone

	Linear indirect	Linear direct	Exponential indirect	Exponential direct	Real GSI
E2	0.63	0.62	0.48	0.46	0.77
T	0.71	0.72	0.62	0.62	0.88
11‐KT	0.81	0.81	0.80	0.78	0.74
FSH	0.50	0.50	0.34	0.33	0.32
LH	0.77	0.76	0.60	0.58	0.57
MIH	0.48	0.46	0.51	0.48	0.27

## Discussion

GSI has been a standard method for evaluating progress in sexual maturation of salmonids. Here, a new, noninvasive method that uses ultrasound for estimating GSI in females from 1 year before ovulation has been presented. We found that ultrasound‐based ovary length measurements can be used to monitor GSI in maturing females and it can give an accurate picture of maturational progress in combination with oocyte size observations by ultrasound.

The first application of ultrasound in a salmonid fish, was aimed at sex differentiation for broodstock management and estimating amount of sexual maturation in harvest fish (Martin et al. 1983; Reimers et al. 1987). Mattson (1991) expanded this concept by measuring ovary diameter using ultrasound, and suggested that gonad length and diameter could be used to estimate gonad weight. Models have been developed for estimating GSI in females, by combining area measurements in cross‐sectional images of ovaries and external length measurements (European eel; Bureau du Colombier et al. 2015), or by converting calculated ovarian volume to weight through stage specific density factors (pallid sturgeon, *Scaphirhynchus albus*; Albers et al. 2013). These methods require detailed measurements and calculation after ultrasound examination for GSI estimation, and are thus little applicable for real‐time GSI estimation when working with live fish. In this study, we found two models for real‐time GSI estimation in live females, using ultrasound‐based ovary length measurements that are performed in a few seconds. GSI can either be modeled directly from ultrasound measured left ovary length, or indirectly by combining modeled total ovary weight with female body weight measurements. Linear and exponential models for both direct and indirect GSI estimation were developed in this study. Direct and indirect linear models performed equally well after freshwater transfer. Both exponential models have good performance during seawater phase when ovaries are short, but are not recommended after freshwater transfer due to an increasing overestimation of GSI with increasing ovary lengths. Both approaches provide real‐time GSI results that can be used instantly to grade females according to their maturational status, and examinations can be performed on the same female repeatedly. Ultrasound equipment has lately gone from large stationary scanners to portable units, therefore this technology has become more accessible for field studies and other applications. This noninvasive approach to maturation monitoring would improve animal welfare according to two of the three Rs in the guidelines for animal welfare. Eliminating the need to sacrifice fish or the use of other invasive methods, and the possibility to examine the same individual repeatedly, reduces the number of fish needed for examination and refines maturation monitoring (Russell and Burch 1959).

The observed extensive oocyte development during seawater phase did not correlate with performed monthly GSI and sex hormone measurements. Used independently to histological studies, these methods should therefore be applied with care when monitoring Atlantic salmon female maturation in seawater.

Ultrasound could not clearly visualize changes in oocyte development in the last 12 to 8 months before ovulation, since the only observation was an increase in diameter of a uniformly structured ovary. From 7 months before ovulation and onwards, tertiary yolk oocytes were distinguishable in ultrasound images. When individual females were rearranged according to oocyte development stage, strong correlations between ultrasound estimated GSI, plasma sex hormone levels and oocyte development was revealed. Females with more developed oocytes, that is, tertiary yolk oocytes, showed higher ultrasound estimated GSI and level of hormones involved in inducing vitellogenesis, such as FSH and E2, indicating that hormonal processes had been initiated earlier than what could be observed from the monthly hormone profiles. This is in accordance with findings of Taranger et al. (1999), where increased plasma E2 levels were observed in January in females that matured the following autumn. Thus, ultrasound examination could replace sacrifice for histology sampling and less invasive blood sampling in Atlantic salmon females.

All females in this study matured normally although some unexpected findings included the presence of vitellogenic oocytes in November 2014 (Fig. 3), 8 months before significant changes in GSI (Fig. 1 in Figs. S2 and S3), and sex hormones levels were observed (Fig. 2 in Figs. S2 and S3). This is somewhat earlier than expected compared to other studies in Atlantic salmon females, where secondary growth phase oocytes were observed equally early under similar photoperiod treatment, but not any vitellogenic oocytes (Taranger et al. 1998, 1999; Andersson et al. 2009). Recently, high expression of vitellogenin receptors in ovaries from females during the same period have been observed, supporting our observation (Andersen et al. 2017) The early development of vitellogenic oocytes found here appears to be more similar to observations in rainbow trout (*Oncorhynchus mykiss*; Sumpter et al. 1984).

Low levels of 11‐KT were described for the first time in maturing Atlantic salmon females (Fig. 2 in Figs. S2 and S3). Studies in short‐finned eel (*Anguilla australis*) and Atlantic cod (*Gadus morhua* L.) have indicated that 11‐KT stimulates previtellogenic oocyte growth (Lokman et al. 2007; Kortner et al. 2009a,b), and it has also been shown to induce silvering‐related changes in female short‐finned eels (Rohr et al. 2001). Fitzpatrick et al. (1986) reported that female Coho salmon (*Oncorhynchus kisutch*) with more advanced maturation status, had higher plasma levels of 11‐KT than less mature females, although nonsignificant. In our study, levels of 11‐KT in females were very low (1–2 ng/mL) compared to other steroids (E2: ~40 ng/mL). However, there was a significant step‐wise increase and decrease during the freshwater phase toward ovulation. A role of 11‐KT in previtellogenic oocytes could not be confirmed in this study, as levels remained low during the short period before yolk accumulation started in November 2014. On the other hand, we observed increasing levels of 11‐KT closer to ovulation, suggesting that 11‐KT may be involved in regulation or stimulation of final maturation and ovulation in Atlantic salmon.

Ultrasound technology is well suited to substitute traditional invasive methods during sexual maturation monitoring in Atlantic salmon broodfish populations. Ultrasound‐based ovary length measurements correlated strongly with GSI and sex hormone levels. In addition, oocyte size observations by ultrasound corresponded well to histologically identified stages of oocyte development. These methods offer a possibility to reduce stress and improve animal welfare in broodstock management of both wild and farmed Atlantic salmon populations.

## Conflict of Interest

All authors declare no conflict of interest.

## Data Accessibility

## Supporting information




**Figure S1.** Rearing conditions. Temperature (blue), and natural (black) and artificial (dashed line) light profiles during seawater and freshwater phases. Horizontal boxes represent seawater and freshwater phases.

**Figure S2.** Gonado‐somatic index (GSI) in female Atlantic salmon during the last year before ovulation. Samplings from September 2014 through July 2015 are monthly, while August and September 2015 samplings are weekly. Horizontal bars at the top represents changes in environment conditions. Data are mean ± SEM. Letters indicate significant differences, *P* < 0.05. Break in x axis indicates sampling frequency changing from monthly to weekly.

**Figure S3.** Profiles of plasma sex steroids in female Atlantic salmon during the last year before ovulation. Samplings are monthly from September 2014 through July 2015, and then weekly from there. (A) oestradiol, (B) testosterone, (C) luteinizing hormone, (D) follicle stimulating hormone, (E) 11‐keto testosterone hormone, (F) maturation inducing hormone. Data are mean ± SEM. Letters indicate significant differences, *P* < 0.05. Horizontal bars at the top represents changes in environment conditions. Break in x axis indicates sampling frequency changing from monthly to weekly.

**Table S1.** Mean fish weight (±SEM) and *K* factor (±SEM) for all samplings. Number of samples analyzed for sex hormone concentration and histology for each sampling. 11‐KT, 11‐keto testosterone; E2, oestradiol; FSH, follicle‐stimulating hormone; LH, luteinizing hormone; MIH, maturation inducing hormone, T, testosterone.

**Table S2.** Histological classification of oocytes in sections from Atlantic salmon ovaries (Taranger et al. 1999; Andersson et al. 2009).
