# Supplementary figures and images for: Ultrasound as a noninvasive tool for monitoring reproductive physiology in female Atlantic salmon (Salmo salar)
Source: Physiol Rep. 2018 May 6;6(9):e13640. doi: 10.14814/phy2.13640 (PMC5936688; doi:10.14814/phy2.13640)

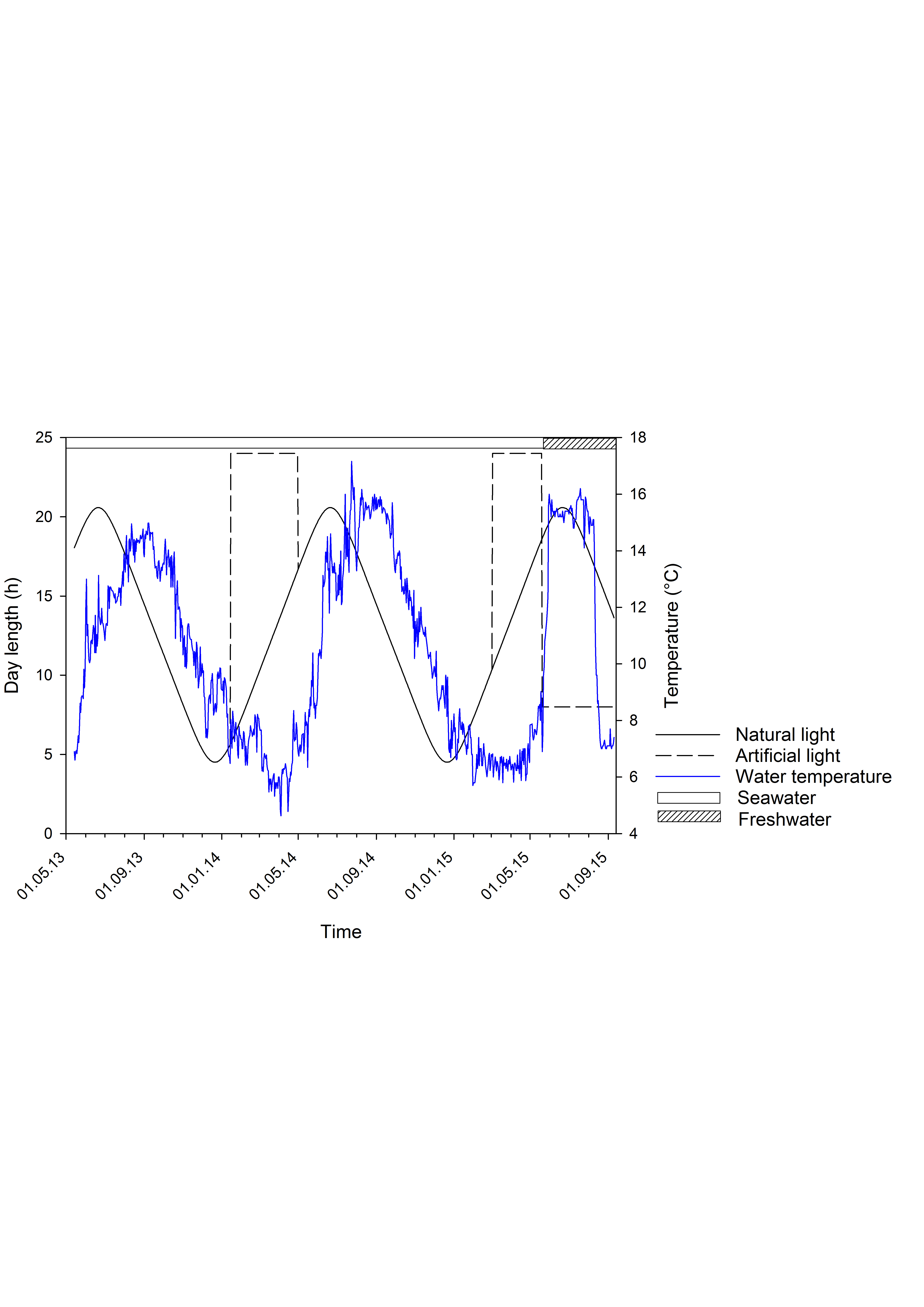

Supplement: Supplementary file 1 — Figure S1. Rearing conditions. Temperature (blue), and natural (black) and artificial (dashed line) light profiles during seawater and freshwater phases. Horizontal boxes represent seawater and freshwater phases. [file PHY2-6-e13640-s001.JPG]

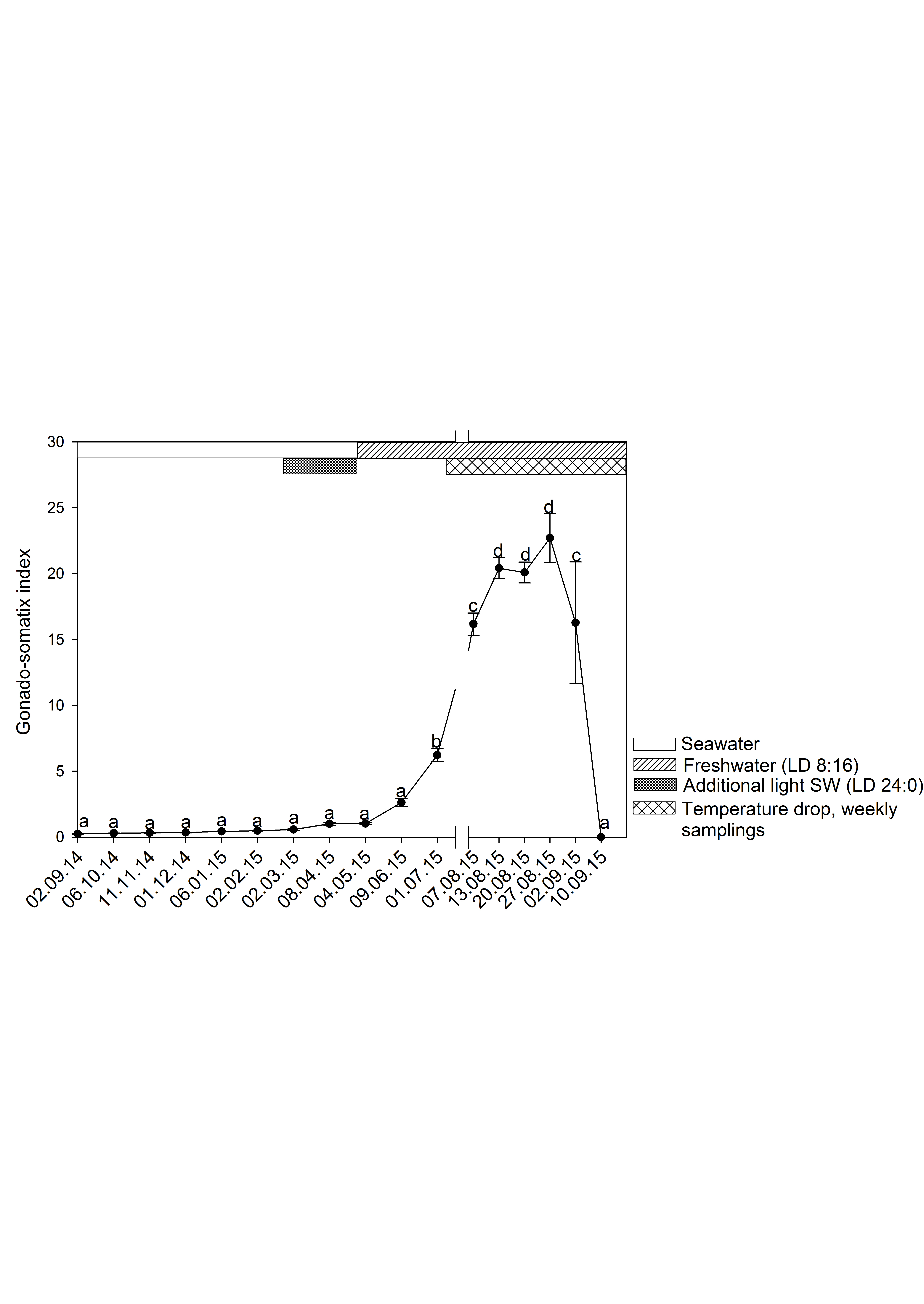

Supplement: Supplementary file 2 — Figure S2. Gonado‐somatic index (GSI) in female Atlantic salmon during the last year before ovulation. Samplings from September 2014 through July 2015 are monthly, while August and September 2015 samplings are weekly. Horizontal bars at the top represents changes in environment conditions. Data are mean ± SEM. Letters indicate significant differences, P < 0.05. Break in x axis indicates sampling frequency changing from monthly to weekly. [file PHY2-6-e13640-s002.JPG]

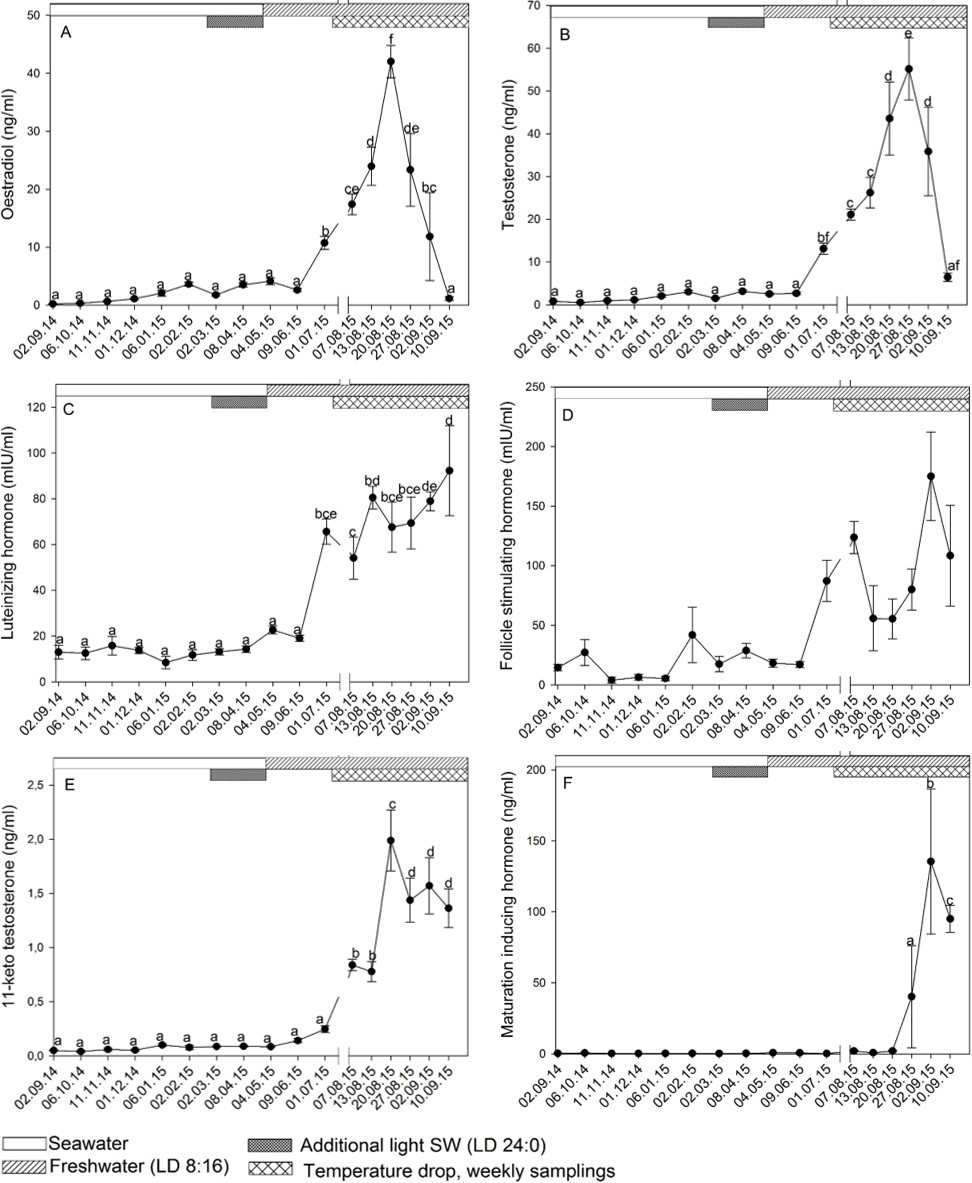

Supplement: Supplementary file 3 — Figure S3. Profiles of plasma sex steroids in female Atlantic salmon during the last year before ovulation. Samplings are monthly from September 2014 through July 2015, and then weekly from there. (A) oestradiol, (B) testosterone, (C) luteinizing hormone, (D) follicle stimulating hormone, (E) 11‐keto testosterone hormone, (F) maturation inducing hormone. Data are mean ± SEM. Letters indicate significant differences, P < 0.05. Horizontal bars at the top represents changes in environment conditions. Break in x axis indicates sampling frequency changing from monthly to weekly. [file PHY2-6-e13640-s003.jpg]
