# Supplementary material for: Ultrasound as a noninvasive tool for monitoring reproductive physiology in female Atlantic salmon (Salmo salar)
Source: Physiol Rep. 2018 May 6;6(9):e13640. doi: 10.14814/phy2.13640 (PMC5936688; doi:10.14814/phy2.13640)
Supplement: Supplementary file 4 — Table S1. Mean fish weight (±SEM) and K factor (±SEM) for all samplings. Number of samples analyzed for sex hormone concentration and histology for each sampling. 11‐KT, 11‐keto testosterone; E2, oestradiol; FSH, follicle‐stimulating hormone; LH, luteinizing hormone; MIH, maturation inducing hormone, T, testosterone. [file PHY2-6-e13640-s004.docx]

Table S1. Mean fish weight (± S.E.M) and K factor (± S.E.M) for all samplings. Number of samples analysed for sex hormone concentration and histology for each sampling. 11-KT, 11-keto testosterone; E2, oestradiol; FSH, follicle-stimulating hormone; LH, luteinizing hormone; MIH, maturation inducing hormone, T, testosterone.

| Sampling date  (n)^1^ | Fish weight (kg)  (mean ± S.E.M)^2^ | K factor  (mean ± S.E.M) | 11-KT, E2, LH,  MIH, T (n) | FSH (n) | Histology (n) |
| --- | --- | --- | --- | --- | --- |
| 02.09.14 (20) | 5.75 ± 0.23 | 1.20 ± 0.02 | 6 | 3 | 6 |
| 06.10.14 (20) | 6.08 ± 0.16 | 1.16 ± 0.02 | 6 | 5 | 6 |
| 11.11.14 (20) | 7.22 ± 0.20 | 1.28 ± 0.02 | 6 | 2 | 6 |
| 01.12.14 (20) | 8.12 ± 0.29 | 1.31 ± 0.01 | 6 | 5 | 6 |
| 06.01.15 (20) | 8.36 ± 0.40 | 1.26 ± 0.03 | 6 | 5 | 6 |
| 02.02.15 (20) | 9.90 ± 0.38 | 1.42 ± 0.04 | 6 | 4 | 6 |
| 02.03.15 (19) | 10.93 ± 0.45 | 1.44 ± 0.04 | 10 | 9 | 10 |
| 08.04.15 (15) | 12.56 ± 0.41 | 1.50 ± 0.01 | 10 | 10 | 10 |
| 04.05.15 (20) | 11.60 ± 0.31 | 1.39 ± 0.08 | 10 | 10 | 10 |
| 09.06.15 (20) | 11.09 ± 0.35 | 1.30 ± 0.02 | 10 | 10 | 10 |
| 01.07.15 (20) | 10.55 ± 0.36 | 1.30 ± 0.03 | 20 | 19 | 20 |
| 07.08.15 (5) | 11.43 ± 0.75 | 1.28 ± 0.03 | 5 | 5 |  |
| 13.08.15 (5) | 10.26 ± 0.41 | 1.22 ± 0.03 | 5 | 5 |  |
| 20.08.15 (5) | 9.88 ± 0.75 | 1.17 ± 0.05 | 5 | 5 |  |
| 27.08.15 (5) | 10.76 ± 0.43 | 1.16 ± 0.03 | 5 | 5 |  |
| 02.09.15 (5) | 10.48 ± 0.55 | 1.21 ± 0.05 | 5 | 5 |  |
| 10.09.15 (5) | 10.21 ± 0.54 | 1.18 ± 0.02 | 5 | 5 |  |

^1^In March and April 2015 fewer females were sampled due to weather conditions.

^2^ There was significant increase in body weight between October and November 2014, and monthly from January to April 2015.
