# Supplementary material for: Ultrasound as a noninvasive tool for monitoring reproductive physiology in female Atlantic salmon (Salmo salar)
Source: Physiol Rep. 2018 May 6;6(9):e13640. doi: 10.14814/phy2.13640 (PMC5936688; doi:10.14814/phy2.13640)
Supplement: Supplementary file 5 — Table S2. Histological classification of oocytes in sections from Atlantic salmon ovaries (Taranger et al. 1999; Andersson et al. 2009). [file PHY2-6-e13640-s005.docx]

Table S2. Histological classification of oocytes in sections from Atlantic salmon ovaries (Taranger *et al.*, 1999; Andersson *et al.*, 2009).

| Oocyte stage | Description | Phase |
| --- | --- | --- |
| Chromatin nucleolus stage | Small oocyte with visible nucleolus and chromatin threads | Primary growth phase |
| Perinucleolar stage | Blue staining nucleoli visible around the nucleus | “ |
| Cortical alveoli stage | Cortical alveoli around the periphery (and in center, depending on section) of oocyte | Secondary growth phase |
| Oil droplet stage | Oil droplets in the center of the oocyte | “ |
| Primary yolk stage | Small amounts of yolk appear in the periphery of the oocyte | True vitellogenesis |
| Secondary yolk stage | Yolk globule fills more of the oocyte, the globule increases in size | “ |
| Tertiary yolk stage | Whole oocyte filled with large yolk globules | “ |
| Atretic oocyte | Breakdown of oocyte content and membranes, increasing folding of follicular layers | Other |
| Other tissues | Blood vessels, connective tissue, adipose tissue |  |
